# Opening of Medical Colleges

**Published:** 1853-11

**Authors:** 


*Opening of Medical Colleges.*—The portals of our medical schools are now again opened for the reception of students. The classes in attendance are nowhere very large, but there seems to be some reaction in favor of the provincial schools. Thus, Albany and Pittsfield exceed their usual numbers, while in the great cities there is a falling off. The College of Physicians and Surgeons at New York, has, however, an increased class. The University of Louisville, where our senior is now lecturing, is favored with continued and deserved prosperity.

At the Buffalo medical school, the general term has now commenced with a larger class present at its opening than last year. Large accessions to it are anticipated. The new Professor of Theory and Practice, Dr. Thomas F. Rochester, has, during the preliminary term, delivered very acceptable cliniques; and his opening lectures at the general term, indicate that the high expectations which have been held of his excellence in his department, are to be fully realized. S. B. H.

